# Knowledge and Support for Antimicrobial Stewardship Does Not Necessarily Translate into Good Practice: Survey in a Tertiary Hospital in Moldova, May–June 2024

**DOI:** 10.3390/antibiotics14121180

**Published:** 2025-11-21

**Authors:** Nadejda Morărescu, Pawel Stefanoff

**Affiliations:** 1 National Agency for Public Health (NAPH), MD-2028 Chișinău, Moldova; 2 Mediterranean and Black Sea Programme for Intervention Epidemiology Training (MediPIET), European Centre for Disease Prevention and Control (ECDC), 17183 Stockholm, Sweden

**Keywords:** knowledge, attitude, practice, antimicrobial stewardship, healthcare professionals, Moldova

## Abstract

Background/Objectives: Antimicrobial resistance (AMR) is an increasing problem globally, largely driven by the overuse and misuse of antibiotics. In Moldova, physicians are frequently not following recommendations regarding the use of antimicrobials. Despite the adoption of a national strategy to combat AMR, information on healthcare professionals’ knowledge, attitudes, and practices (KAP) related to antimicrobial stewardship (AMS) remains limited. This study aimed to assess KAP related to AMS among staff at a tertiary hospital. Methods: We surveyed employees of the Republican Clinical Hospital between 20 May and 30 June 2024. We interviewed doctors, nurses, pharmacists, clinical pharmacologists, and epidemiologists using a structured, self-administered questionnaire. The instrument assessed their KAP regarding antimicrobial stewardship. We analyzed the data collected using descriptive statistics and tests of association. Results: Among 138 participants, 65% were female and 54% were nurses. A high proportion demonstrated good knowledge (87%) and a positive attitude (78%) towards AMS. However, only 31% reported good stewardship-related practices. Significant associations were observed between knowledge and attitude (*p* < 0.001), and between knowledge and practice (*p* = 0.013). No significant association was found between attitude and practice (*p* = 0.160). Conclusions: These findings reveal a disconnect between knowledge, attitudes, and practical implementation of AMS principles. While healthcare professionals were knowledgeable and well-intentioned, practice remains inconsistent. This suggests that effective AMS interventions must combine individual training with structural support at the institutional level. Tailored strategies should address both general awareness and hospital-specific factors contributing to AMR.

## 1. Introduction

In May 2015, the World Health Assembly’s endorsed the Global Action Plan on Antimicrobial Resistance (AMR) [1], recognizing antimicrobial resistance (AMR) as a global threat to public health. WHO acknowledged the overuse and misuse of antimicrobials as a main driver for the development of resistance, as well as the need to optimize antimicrobial use.

Antimicrobial drugs have been widely used in human medicine for more than 50 years either as prophylaxis or treatment, with tremendous benefits for public health. Unfortunately, their widespread, inappropriate, and often unnecessary use has led to the emergence of antimicrobial-resistant microorganisms [2]. According to a World Health Organization (WHO) report, more than 50% of all antibiotics are incorrectly prescribed, sold, or dispensed [3].

Reducing inappropriate antibiotic use is considered the most effective approach to manage its adverse effects [4]. While understanding of the consequences of antibiotic misuse is increasing, overprescribing remains common, driven largely by patient demand, time pressure on clinicians, and diagnostic uncertainty [2].

Stewardship is defined as “the careful and responsible management of something entrusted to one’s care” [5]. In healthcare settings, this concept is operationalized through “antimicrobial stewardship programs” (ASP), which aim to optimize the use of antimicrobials [6]. Over time, stewardship has also come to refer more broadly to the governance of health systems, with an emphasis on guiding actions at both national and global levels [7].

In Moldova, the National Program for Antimicrobial Resistance Surveillance and Control 2023–2027 highlights the importance of increasing awareness of antimicrobial resistance (AMR) among healthcare professionals. One of its key objectives is to strengthen health education and promote the rational use of antimicrobials. Communication and awareness-raising activities are planned at all levels to improve knowledge and stimulate behavior change among healthcare workers [8].

Supporting the need for such efforts, a 2018 Point Prevalence Survey (PPS) on healthcare-associated infections and antimicrobial use [9], conducted in 67 hospitals out of approximately 90 medical institutions operating in Moldova [10] revealed that antibiotics were mostly used for treatment (74%) and for prolonged surgical prophylaxis exceeding one day.

Despite the implementation of national initiatives addressing antimicrobial resistance, there is limited evidence on how healthcare professionals in Moldova perceive and engage with antimicrobial stewardship. To address this gap, we conducted a cross-sectional study assessing the knowledge, attitudes, and practices of medical staff working in a tertiary-level hospital. The findings aim to inform future antimicrobial stewardship interventions at the institutional and national levels.

## 2. Results

### 2.1. Socio-Demographic Characteristics of Study Participants

A total of 138 healthcare professionals participated in the study; 65% were women. Most respondents were nurses (54%), mostly employed in surgery (50%) or intensive care (31%). Participants represented a range of ages and years of experience (Table 1).

### 2.2. Education and Training Regarding Antimicrobial Stewardship

Most respondents (88%) supported mandatory AMS education. While 68% reported institutional support for continuous training, only 62% felt adequately prepared to apply stewardship principles, indicating room for improvement in practical and institutional support.

### 2.3. Basic Knowledge of Healthcare Professionals on AMS

Respondents demonstrated a strong understanding of AMS-related concepts: 95% acknowledged that inappropriate antibiotic use drives resistance, and 93% agreed it reduces treatment effectiveness. However, only 43–50% reported familiarity with specific AMS terms like “de-escalation” or “watchful waiting”.

Most respondents identified irrational antibiotic use in the general population and in healthcare settings as the main drivers of AMR. Detailed responses are summarized in Additional file 1: Supplementary Table S1. Perceived causes of irrational antimicrobial use are summarized in Additional file 2: Supplementary Figure S1.

### 2.4. Attitude of Healthcare Professionals Towards Antimicrobial Resistance and Stewardship

The majority (91%) viewed AMR as a global threat and recognized the value of AMS programs (90%). Shared responsibility in antibiotic use and patient education were also strongly supported by respondents.

### 2.5. Practices of Healthcare Professionals Related to Antimicrobial Resistance and Stewardship

Respondents reported adherence to several key AMS practices; however, overall practice scores were lower than knowledge and attitude scores, including switching from intravenous to oral antibiotics when appropriate and using microbiology results to guide treatment (85%). However, 77% supported the use of broad-spectrum antibiotics to counter resistance, which may indicate gaps in understanding appropriate antibiotic selection.

### 2.6. KAP Scores

Overall, 87% (*n* = 120) of respondents demonstrated good knowledge, 78% (*n* = 108) had a positive attitude, and 31% (*n* = 43) reported good AMS-related practices (Table 2).

A total of 18 respondents (13%) had poor knowledge, while 30 (22%) demonstrated a negative attitude. Poor implementation of AMS-related practices was observed in 95 respondents (62%).

There were no major differences in knowledge or attitude scores across age groups or years of professional experience (Table 3). However, a decline in practice scores was observed among healthcare workers with the longest experience. Employees from surgical wards showed the lowest mean scores across all three domains: knowledge, attitude, and practice, with the difference being most evident for practice. These findings suggest that while knowledge and attitudes remained relatively consistent across subgroups, longer experience and departmental context appeared to influence the consistent application of stewardship principles in daily clinical work (Table 3).

### 2.7. Association Between Knowledge, Attitude and Practice

We observed significant associations between knowledge and attitude (*p* < 0.001), and between knowledge and practice (*p* = 0.013), indicating that participants with good knowledge were more likely to demonstrate a positive attitude and report good stewardship-related practices. We did not find significant association between attitude and practice (*p* = 0.160) (Table 4). These results support the hypothesis that knowledge plays a key role in shaping both attitudes and behaviors related to AMS. However, the lack of significant association between attitude and practice may suggest existence of institutional barriers influencing the translation of beliefs into action.

## 3. Discussion

This study assessed the knowledge, attitudes, and practices (KAP) related to antimicrobial stewardship (AMS) among healthcare professionals at the largest tertiary hospital in Moldova. The main finding of this study is the clear disconnect between healthcare professionals’ knowledge and attitudes versus their reported practices regarding antimicrobial stewardship.

First, most respondents demonstrated good knowledge and supportive attitudes towards AMS. Second, reported practices remained considerably lower despite high awareness. Third, bivariate analysis confirmed that knowledge significantly influenced both attitude and practice, but attitude alone did not translate into improved practice. Comparable findings were reported in Ghana, where a multicenter cross-sectional survey across six public hospitals found that healthcare professionals exhibited favorable knowledge and attitudes but limited adherence to AMS practices, illustrating that awareness alone does not guarantee consistent stewardship behaviors [11].

Approximately 87% of respondents demonstrated good knowledge and 78% expressed a positive attitude towards AMS. These findings suggest that existing educational initiatives and awareness efforts may have a positive effect. Similarly, high levels of knowledge and supportive attitudes have been documented in other settings, including Ethiopia [12]. However, our results also reveal neutral perceptions regarding institutional resource availability and limited confidence in applying AMS principles. These mixed responses highlight that while basic awareness is present, implementation capacity may remain underdeveloped. This observation aligns with the Ghana study, which also emphasized the need for robust hospital AMS committees, continuous professional training, and monitoring systems to strengthen the link between knowledge and action [11].

Differences across professional experience and professional factors may also influence the translation of stewardship principles into practice. Previous studies have shown that more experienced healthcare workers often relied on established clinical routines, which could limit adherence to AMS guidelines [13]. Similarly, professionals working in high-pressure departments such as surgery reported lower compliance with AMS interventions, largely due to workload and urgent decision-making demands [14]. These findings highlight the importance of department-specific strategies and mentorship programs to support practical implementation of stewardship principles across all staff levels. This observation is consistent with recent studies from surgical departments, where lower adherence to antimicrobial stewardship practices has been observed compared with medical wards [15].

Nurses made up the largest proportion of respondents, consistent with their high representation among hospital staff. As evidenced in several studies, nurses can play a crucial role in AMS implementation, particularly through infection prevention and control practices and early warning for inappropriate prescribing. This pattern mirrors the Ghanaian data, where the predominance of nurses and inconsistent institutional support were identified as key barriers to applying stewardship principles in clinical settings [11].

Despite positive knowledge and attitudes, only 31% of participants reported good stewardship practices. This disconnect is consistent with other published findings [12], which noted a gap between awareness and implementation. Comparable findings were also reported by Kiani et al. among medical residents in Iran, where high awareness of rational antibiotic use coexisted with limited familiarity and engagement in antimicrobial stewardship programs, underscoring that knowledge alone is insufficient to ensure consistent stewardship practices [13]. In contrast, a study from Sudan [16] reported low knowledge level accompanying poor practice, suggesting that different institutional context influence both awareness and behavior. One possible explanation for our findings is that practical barriers, such as lack of institutional support, unclear protocols, or insufficient feedback mechanisms, limit the translation of attitudes into action. This is further supported by the finding that only 62% of respondents felt adequately prepared to apply AMS principles, despite 68% reporting institutional support for continuous training. These findings underline the importance of not only education but also system-level interventions to facilitate behavior change.

The significant associations observed between knowledge and both attitude (*p* < 0.001) and practice (*p* = 0.013) reinforce the idea that strengthening knowledge is foundational to changing behaviors. However, the lack of significant correlation between attitude and practice suggests that favorable attitudes alone are insufficient. This highlights a critical implementation gap: even motivated professionals may struggle to apply AMS practices without clear institutional support, structured protocols, and regular feedback mechanisms.

Our investigation had several limitations. First, the study was conducted in a single tertiary hospital, which may limit generalizability to other settings. Second, the cross-sectional design did not permit assessing changes over time or causal inferences. Third, the self-administered questionnaire could introduce social desirability or response bias. Nonetheless, efforts were made to ensure anonymity and encourage honest reporting. Finally, while we developed composite scores to quantify KAP domains, these scores were based on self-reported responses, which may not fully reflect actual behaviors.

In addition, the post-COVID-19 period has been characterized by widespread inappropriate antibiotic use, which has further accelerated concerns about antimicrobial resistance [17]. This reinforces the urgency of strengthening hospital antimicrobial stewardship to close the documented attitude–practice gap in our setting.

## 4. Materials and Methods

### 4.1. Study Area and Study Period

The study was conducted in the Republican Clinical Hospital in Chisinau from 20 May to 30 June 2024. In 2024, this 767-inpatient-bed hospital, the largest in Moldova, had various departments, including therapeutic, surgical, anesthesiology and intensive care, pharmacy & clinical pharmacology, as well as the epidemiological health service.

### 4.2. Study Design

We conducted a cross-sectional study to investigate healthcare workers’ knowledge, attitudes, and practices regarding antimicrobial stewardship. This study was designed and reported in accordance with the STROBE (Strengthening the Reporting of Observational Studies in Epidemiology) guidelines for cross-sectional studies [18].

### 4.3. Study Population

The target population included all healthcare professionals employed in selected departments during the study period and who met the eligibility criteria. We included healthcare workers who met the eligibility criteria:

- Employed at least since December 2023.
- Worked in one of the following departments: intensive care unit (ICU), surgery, therapy, pharmacy and clinical pharmacology, or the epidemiological health service.
- Belonged to one of the following professions: medical doctor, nurse, pharmacist, clinical pharmacologist, or epidemiologist.

We excluded employees who did not consent for participating in the study.

### 4.4. Sample Size and Sampling Technique

We tried to interview all employees of the selected departments. In May 2024, 1502 were employed in these departments. To determine the minimum sample size, we utilized the simple random sample size calculation, using formula with a 98% confidence interval, assuming a 50% prevalence, 10% margin of error. To calculate the minimum sample size required, we used the standard formula for estimating a population proportion:

n = Z^2^ × P(1 − P)/D^2^,

where n is the sample size, Z is the z-score for a 98% confidence level (2.33), P is the expected proportion (set at 0.5 for maximum variability), and D is the margin of error (0.10).

Based on these parameters, the required sample size was 125. Subsequently, we allocated various professions proportionally to the size of each professional group. This approach ensured representation across professional categories and adequate power for subgroup analysis.

### 4.5. Study Variables

- Demographic variables: Age, Gender, Profession, Department and Years of Experience.
- Outcome variables: Knowledge, Attitude, Practice scores, and overall KAP score.

### 4.6. Data Collection Instrument

The data collection instrument was a structured, self-administered questionnaire developed specifically for this study. It was organized into five thematic sections, each targeting a specific aspect of antimicrobial stewardship among healthcare professionals. One interviewer collected all interviews on paper forms in person. The same interviewer entered the forms into a computer database.

### 4.7. Demographic and Professional Characteristics

This section captured basic demographic and professional data, including participants’ age, gender, professional category (e.g., physician, nurse, pharmacist, clinical pharmacologist, epidemiologist), department of employment (e.g., surgery, intensive care, pharmacology), and years of clinical experience.

### 4.8. Education and Training in AMS

This section assessed perceptions related to AMS education and training. Respondents were asked to indicate whether they considered their current education and professional training sufficient to apply AMS principles in clinical settings. Additional items explored their satisfaction with institutional support and available learning resources, how often they engaged in continuing education activities related to AMS, and the extent to which they agreed that AMS training should be mandatory for all healthcare professionals.

### 4.9. Knowledge

This section included five declarative statements addressing the consequences of inappropriate antimicrobial use, such as the development of resistance, adverse effects, treatment failure, and additional financial burden, as well as the preventive role of AMS in healthcare-associated infections. Respondents rated their level of agreement using a five-point Likert scale (from 1—strongly disagree to 5—strongly agree).

### 4.10. Attitudes

Seven statements were used to measure participants’ attitudes toward AMS and antimicrobial resistance (AMR), including the perceived seriousness of AMR as a public health threat, the role of AMS programs in improving care and reducing resistance, shared responsibility for rational prescribing, and the importance of patient education. Respondents rated their level of agreement using a five-point Likert scale (from 1—strongly disagree to 5—strongly agree).

### 4.11. Practices

This section assessed current AMS practices through six items focused on behaviors such as use of microbiological testing, de-escalation strategies, intravenous-to-oral switch, and adherence to environmental hygiene protocols. Additional multiple-choice questions explored institutional-level contributors to AMR, such as prolonged treatment courses, frequent patient transfers, and non-compliance with infection prevention and control measures. Respondents rated their level of agreement using a five-point Likert scale (from 1—strongly disagree to 5—strongly agree).

In addition to the items used for computing KAP scores, the questionnaire included a separate set of questions assessing healthcare professionals’ familiarity with selected AMS-related concepts. These items used a 5-point Likert scale (ranging from “Not at all familiar” to “Very familiar”) and were analyzed descriptively. For reporting, “Familiar” and “Very familiar” responses were merged.

### 4.12. Calculation of Scores

For the calculation of scores, we assigned one point to each question (Table 5). We considered “Agree” and “Completely agree” as positive answer. All the remaining answers, including missing data, were considered negative. We summed the scores within each dimension (5 for knowledge, 7 for attitude, and 6 for practice). We considered knowledge as good when at least 4 of 5 questions (>65%) were correctly answered, the attitude as positive when at least 6 of 7 questions (>75%) concurred with supportive attitude, and practice as good when at least 5 of 6 questions (>70%) indicated compliance.

### 4.13. Data Analysis

The database used for analysis did not contain personal information about respondents or the department involved in the study. For statistical analysis we used IBM SPSS Statistics version 31.0.0.0 (117) and Microsoft Excel 2018. The analysis focused on computing KAP scores and comparing them by demographic and professional characteristics and identifying trends related to antimicrobial stewardship. For clarity in reporting, we merged the response categories “Agree” and “Completely agree” into a single category labeled “correct answer” in the presentation of results. To present the results, we used frequencies, means, standard deviations.

We assessed associations between knowledge, attitude, and practice scores using chi-square and Fisher’s exact tests to identify significant relationships between the three KAP domains. A significance level of *p* < 0.05 was considered statistically significant for all analyses.

### 4.14. Data Quality Assurance

The interviewer noted any missing or unclear information in the comments section. In cases where essential information was not recorded, it was annotated as “missing data”.

### 4.15. Ethical Consideration

To assure compliance with human subject protection principles, the principal investigator ensured that the data collection instruments and protocol of the survey were submitted to the National Ethics Committee for Clinical Studies Review. The survey was initiated only after receiving approval.

## 5. Conclusions

Our findings suggest that while healthcare professionals in a tertiary hospital in Moldova demonstrated good knowledge and supportive attitudes towards AMS, gaps remained in practice implementation. In particular, nearly one in five respondents expressed negative attitudes and more than two-thirds reported poor implementation of AMS-related practices, with lower scores observed among staff from surgical departments. Targeted strategies are needed to bridge this gap, combining continuous education with system-level support, clear protocols, and stronger IPC measures. Addressing these gaps could significantly improve the effectiveness of AMS programs and help contain antimicrobial resistance in Moldova and similar settings.

Based on our findings, we recommend three key actions to strengthen antimicrobial stewardship (AMS) in Moldova’s hospital settings, aligned with the objectives of the National Action Plan on AMR. First, we recommend expanding AMS-focused training, particularly for nurses and early-career professionals, to consolidate the already high levels of knowledge and positive attitudes. Second, we recommend introducing institutional-level support mechanisms, including standard AMS protocols, multidisciplinary collaboration, and regular audit and feedback. Such mechanisms are essential to bridge the gap between awareness and action, particularly in settings where clinical accountability and AMS leadership structures are underdeveloped. Third, we recommend addressing implementation barriers, such as unclear responsibilities and insufficient IPC integration, to ensure that favorable attitudes are translated into actual practice. These steps are necessary to ensure that AMS is consistently applied, not just understood.

Although this study was conducted in a single tertiary hospital in Moldova, its findings are relevant for similar healthcare settings in other low- and middle-income countries, where AMS implementation faces comparable challenges.

## Figures and Tables

**Table 1:** Characteristics of respondents (*n* = 138), survey of healthcare workers, Moldova, May–June 2024.

	Category	Frequency (%)
Sex	Female	90 (65%)
	Male	48 (35%)
Age	20–30	42 (30%)
	31–40	42 (30%)
	41–50	27 (20%)
	51–60	23 (17%)
	>60	4 (2.9%)
Profession	Nurse	74 (54%)
	Medical Doctor	40 (29%)
	Medical resident	19 (13%)
	Clinical Pharmacologist/Pharmacologist	3 (2%)
	IPC specialists	2 (1.4%)
Department	Surgery	69 (50%)
	Intensive care therapy	43 (31%)
	Therapy	21 (15%)
	Pharmacology/clinical pharmacology	3 (2.2%)
	Epidemiological health-service	2 (1.4%)
Years of experience	0–4	41 (30%)
	5–9	24 (17%)
	>9	73 (53%)

**Table 2:** Mean scores for each question and correct responses proportion of respondents (*n* = 138), Moldova, May–June 2024. The domain-level and global average scores are bolded.

Score Components (Questions on Likert Scale) *	Mean Score (SD)	Correct Answers (%)
AMS prevents healthcare-associated infections (1–5)	4.30 (0.76)	121 (87.7)
Inappropriate antibiotic use leads to resistance (1–5)	4.49 (0.65)	131 (94.9)
Inappropriate use increases adverse effects (1–5)	4.41 (0.66)	125 (90.6)
Inappropriate use may reduce treatment effectiveness (1–5)	4.49 (0.65)	129 (93.5)
Inappropriate use adds financial burden for patients (1–5)	4.24 (0.79)	118 (85.5)
**Knowledge score (0–5)**	**4.55 (0.95)**	**120 (86.9)**
AMR is a significant global public health challenge (1–5)	4.43 (0.63)	126 (91.3)
AMR is a problem in your hospital (1–5)	3.98 (0.77)	106 (76.8)
Reducing unnecessary antibiotic use is a shared responsibility (1–5)	4.27 (0.68)	118 (85.5)
AMS programmes are important to control AMR (1–5)	4.28 (0.61)	125 (90.6)
AMS interventions improve patient outcomes (1–5)	4.09 (0.63)	116 (84.1)
Educating patients about proper antibiotic use is part of stewardship (1–5)	4.33 (0.56)	131 (94.9)
Experts are concerned about the consequences of antibiotic overuse (1–5)	4.41 (0.60)	130 (94.2)
**Attitude score (0–7)**	**6.32 (1.31)**	**108 (78.3)**
Cautious antibiotic use may mitigate AMR (1–5)	4.36 (0.56)	131 (94.2)
Broad-spectrum antibiotics help combat AMR compared to narrow- spectrum (1–5)	2.93 (0.81)	35 (25.4)
Microbiology results are essential for patient care (1–5)	4.25 (0.58)	126 (91.3)
Restrictions on antibiotic prescribing could impact patient care (1–5)	3.50 (0.90)	72 (52.2)
Switching from IV to oral antibiotics after 3 days is advisable when justified (1–5)	3.59 (0.93)	79 (57.3)
Ensuring patient room sanitation is crucial (1–5)	4.55 (0.63)	123 (89.1)
**Practice score (0–6)**	**4.16 (1.18)**	**43 (31.2)**
**Overall KAP score (0–18)**	**15.11 (2.92)**	**N/A**

* Likert scale—from 1 (Strongly disagree) to 5 (Strongly agree). SD—standard deviation.

**Table 3:** Comparison of KAP scores by age, experience, and department. Survey of healthcare workers Moldova, May–June 2024.

	*n*	Knowledge Score (0–5)	Attitude Score (0–7)	Practice Score (0–6)	Overall KAP Score (0–18)
Age group (years)					
20–29	36	4.64	6.31	4.39	15.43
30–39	46	4.66	6.22	4.15	15.03
40–49	25	4.32	6.45	4.29	15.40
50+	31	4.50	6.36	3.78	14.59
Professional experience (years)					
<10	59	4.71	6.32	4.34	15.42
10–20	23	4.54	6.55	4.50	15.94
20–30	18	4.72	6.71	4.25	15.75
30–40	17	4.31	6.33	4.17	14.91
40+	21	4.19	5.67	3.29	13.00
Department					
Epidemiological health-service	2	5.00	7.00	6.00	18.00
Intensive Care Department	43	4.65	6.64	4.47	15.74
Pharmacology and clinical pharmacology	3	4.67	6.67	5.00	17.00
Surgery	69	4.48	6.00	3.85	14.37
Therapy	21	4.52	6.45	4.33	16.00

**Table 4:** Associations between knowledge, attitude and practice towards AMS scores, survey of healthcare workers, Moldova, May–June 2024.

Association	Test Used	χ^2^ (df)	*p*-Value	Fisher’s Exact Test
Knowledge and attitude	Pearson Chi-square	44.573 (1)	<0.001	<0.001
Knowledge and practice	Pearson Chi-square	6.223 (1)	0.013	0.017
Attitude and practice	Pearson Chi-square	1.975 (1)	0.160	0.189

**Table 5:** Questions used to calculate KAP scores, survey of healthcare workers, Moldova, May–June 2024.

Item	Statement	KAP Dimension	Possible Scores
301	AMS prevents healthcare-associated infections	Knowledge	0, 1
302	Inappropriate antibiotic use leads to resistance	Knowledge	0, 1
303	Inappropriate use increases adverse effects	Knowledge	0, 1
304	Inappropriate use may reduce treatment effectiveness	Knowledge	0, 1
305	Inappropriate use adds financial burden for patients	Knowledge	0, 1
	Knowledge score		0–5
401	AMR is a significant global public health challenge	Attitude	0, 1
402	AMR is a problem in your hospital	Attitude	0, 1
403	Reducing unnecessary antibiotic use is a shared responsibility	Attitude	0, 1
404	AMS programmes are important to control AMR	Attitude	0, 1
405	AMS interventions improve patient outcomes	Attitude	0, 1
406	Educating patients about proper antibiotic use is part of stewardship	Attitude	0, 1
407	Experts are concerned about the consequences of antibiotic overuse	Attitude	0, 1
	Attitude score		0–7
501	Cautious antibiotic use may mitigate AMR	Practice	0, 1
502	Broad-spectrum antibiotics help combat AMR compared to narrow-spectrum	Practice	0, 1
503	Microbiology results are essential for patient care	Practice	0, 1
504	Restrictions on antibiotic prescribing could impact patient care	Practice	0, 1
505	Switching from IV to oral antibiotics after 3 days is advisable when justified	Practice	0, 1
506	Ensuring patient room sanitation is crucial	Practice	0, 1
	Practice score		0–6

## Data Availability

Data supporting the findings of this study are available within the article and its Supplementary Materials.

## Supplementary Materials

The following supporting information can be downloaded at: https://www.mdpi.com/article/10.3390/antibiotics14121180/s1. Supplementary Table S1: Factors contributing to emergence of antibiotic resistance, survey of healthcare workers, Moldova, May–June 2024. Supplementary Figure S1: Causes of irrational use of antimicrobials, survey of healthcare workers, Moldova, May–June 2024.

## Abbreviations

The following abbreviations are used in this manuscript:

AMR	Antimicrobial Resistance
ASP	Antimicrobial Stewardship
ASP	Antimicrobial Stewardship Program
HAI	Healthcare-Associated Infection
HCPs	Healthcare Professionals
ICU	Intensive Care Unit
IPC	Infection Prevention and Control
KAP	Knowledge, Attitudes, and Practices
MediPIET	Mediterranean and Black Sea Program for Intervention Epidemiology Training
NAPH	National Agency for Public Health
PPS	Point Prevalence Survey
SPSS	Statistical Package for the Social Sciences
STROBE	Strengthening the Reporting of Observational Studies in Epidemiology
WHO	World Health Organization
