# Supplementary material for: Knowledge and Support for Antimicrobial Stewardship Does Not Necessarily Translate into Good Practice: Survey in a Tertiary Hospital in Moldova, May–June 2024
Source: Antibiotics (Basel). 2025 Nov 21;14(12):1180. doi: 10.3390/antibiotics14121180 (PMC12730089; doi:10.3390/antibiotics14121180)
Supplement: Supplementary file 1 [file antibiotics-14-01180-s001.zip › antibiotics-3918724-supplementary/Additional Files/Additional_file_1_Table_S1.pdf]

Supplementary Table S1: Factors contributing to emergence of antibiotic resistance, survey of healthcare workers, Moldova, May-June 2024.

	Factors	Frequency
1	Excessive/irrational use in the general population	109 (79%)
2	Excessive/irrational use in healthcare facilities	105 (76.1%)
3	Irrational dosage/duration selected in treatment	102 (73.9%)
4	Poor - quality of antimicrobials	55 (39.9%)
5	Inadequate infection control in hospitals	51 (37%)
6	Non-compliance with standard precautions	50 (36.2%)
7	Lack of specific antimicrobials	48 (34.8%)
8	Deficiencies in medical diagnosis	45 (32.6%)
9	Antimicrobial use in animals	34 (24.6%)
10	Low immunization rates	31 (22.5%)
11	Weak regulations	25 (18.1%)
