# Supplementary material for: Knowledge and Support for Antimicrobial Stewardship Does Not Necessarily Translate into Good Practice: Survey in a Tertiary Hospital in Moldova, May–June 2024
Source: Antibiotics (Basel). 2025 Nov 21;14(12):1180. doi: 10.3390/antibiotics14121180 (PMC12730089; doi:10.3390/antibiotics14121180)
Supplement: Supplementary file 1 [file antibiotics-14-01180-s001.zip › antibiotics-3918724-supplementary/Additional Files/Additional_file_2_Figure_S1.pdf]

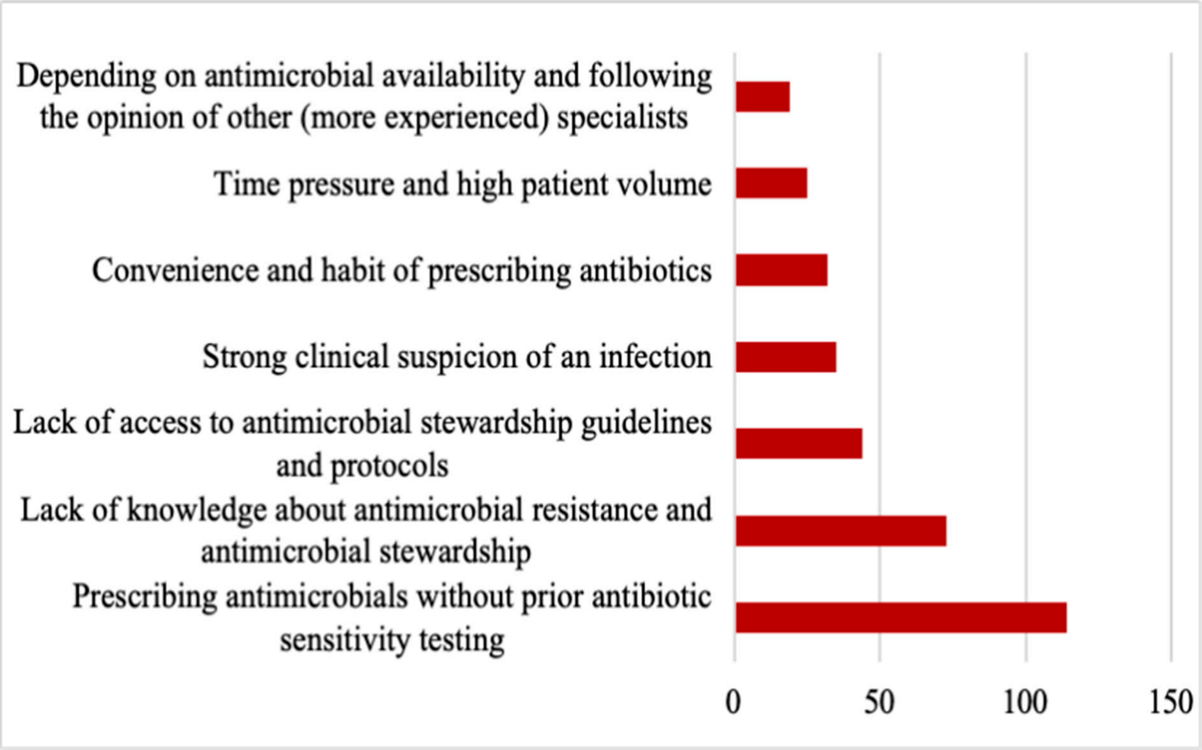

Supplementary Figure S1: Causes of irrational use of antimicrobials, survey of healthcare workers, Moldova, May-June 2024.
